# Text Mining the History of Medicine

**DOI:** 10.1371/journal.pone.0144717

**Published:** 2016-01-06

**Authors:** Paul Thompson, Riza Theresa Batista-Navarro, Georgios Kontonatsios, Jacob Carter, Elizabeth Toon, John McNaught, Carsten Timmermann, Michael Worboys, Sophia Ananiadou

**Affiliations:** 1 National Centre for Text Mining, School of Computer Science, University of Manchester, Manchester, United Kingdom; 2 Centre for the History of Science, Technology and Medicine, University of Manchester, Manchester, United Kingdom; Indiana University, UNITED STATES

## Abstract

Historical text archives constitute a rich and diverse source of information, which is becoming increasingly readily accessible, due to large-scale digitisation efforts. However, it can be difficult for researchers to explore and search such large volumes of data in an efficient manner. Text mining (TM) methods can help, through their ability to recognise various types of semantic information automatically, e.g., instances of concepts (places, medical conditions, drugs, etc.), synonyms/variant forms of concepts, and relationships holding between concepts (which drugs are used to treat which medical conditions, etc.). TM analysis allows search systems to incorporate functionality such as automatic suggestions of synonyms of user-entered query terms, exploration of different concepts mentioned within search results or isolation of documents in which concepts are related in specific ways. However, applying TM methods to historical text can be challenging, according to differences and evolutions in vocabulary, terminology, language structure and style, compared to more modern text. In this article, we present our efforts to overcome the various challenges faced in the semantic analysis of published historical medical text dating back to the mid 19^th^ century. Firstly, we used evidence from diverse historical medical documents from different periods to develop new resources that provide accounts of the multiple, evolving ways in which concepts, their variants and relationships amongst them may be expressed. These resources were employed to support the development of a modular processing pipeline of TM tools for the robust detection of semantic information in historical medical documents with varying characteristics. We applied the pipeline to two large-scale medical document archives covering wide temporal ranges as the basis for the development of a publicly accessible semantically-oriented search system. The novel resources are available for research purposes, while the processing pipeline and its modules may be used and configured within the Argo TM platform.

## Introduction

### Background

Large-scale efforts to digitise vast volumes of historical text are making it increasingly feasible for researchers to use their computers to access, search and explore a wealth of information that was previously only available in printed form. Whilst the wide time spans covered by the digitised texts provide significant scope to study historical change, the amount of data available can be overwhelming. The digitisation of medical journals and public health reports presents new opportunities for medical historians, e.g., to carry out analyses over extended periods of time. However, with this come the challenges of dealing with the changes in the names of diseases and how they were understood. For example, the most common cause of death in the nineteenth century, tuberculosis of the lung, was known successively by its whole body symptoms–*consumption* or *phthisis* (wasting); its pathology—*pulmonary tuberculosis*; and by its cause–*TB* (*Tubercle bacillus*) [1].

More generally, the information needs of historians of medicine usually revolve around *concepts*, e.g., people, places, diseases, drugs, symptoms, and larger information chunks or *relationships* that involve concepts. These include descriptions of which symptoms are caused by specific diseases, which drugs can have an effect on the treatment of a condition, etc. However, typical keyword-based search lacks the expressive power to retrieve sets of documents that specifically correspond to such needs. On the one hand, it can be difficult to retrieve *all* documents that are relevant to a given information requirement, whilst on the other hand, it is frequently the case that many irrelevant documents will be included amongst the results returned by a keyword query.

In terms of the difficulties faced in trying to retrieve all documents relevant to a query, a major problem is that a given concept may be referred to in text in multiple ways. Variations may include synonyms (e.g., *cancer* vs. *tumour* vs. *neoplasm*), spelling variations (*tumour* vs. *tumor)*, abbreviations (*tuberculosis* vs. *TB*), etc. Using standard keyword queries, a researcher must try to enumerate as many as possible of these potential variations, in order to ensure that potentially interesting documents are not overlooked. For long-spanning historical archives, the fact that such variations are subject to change over time can add to the complexity of searching. As discussed above, the terms *tuberculosis*, *phthisis* and *consumption* may (in appropriate contexts) all refer to the same medical condition. However, the latter two terms are normally only used in much older texts. Given the different levels and types of expertise of researchers, it cannot be assumed that they will be aware of all historical variants of a concept of interest.

Conversely, the problem of minimising the number of irrelevant documents that are included within search results is partly complicated by the fact that many words can have multiple meanings, e.g., whilst *consumption* can refer to a disease, this will only be the case in certain contexts, and mostly within a specific time period. Thus, its use as a query term will also return documents where it has other meanings (e.g., ingestion of food and drink).

An additional issue is that keyword-based search cannot be used effectively to restrict search results to just those in which concepts of interest are only mentioned in the context of a relevant *relationship* of interest. As an example, consider that a researcher is interested in finding concepts that correspond to *causes* of tuberculosis. Just as there are various possible ways in which tuberculosis could be mentioned in text, there are also many means of expressing causality, including words and phrases such as *cause*, *due to*, *result of*, etc. Although a researcher could try to formulate a query incorporating multiple variant expressions for both tuberculosis and causality, keyword-based queries do not allow the specification of how different query terms should be *linked* to each other. Thus, in the documents retrieved, there is no guarantee that search terms will even occur within the same sentence and if they do, the nature of the relationship may not be the one that is required. For example, retrieved documents may talk about things *caused by* tuberculosis rather than *causes of* tuberculosis.

Text mining (TM) techniques can help to provide solutions to issues such as the above, in terms of their ability to automatically detect various aspects of the structure and meaning of text. Different TM tools can offer the following relevant functionalities:

- Identifying and semantically classifying *named entities* (NEs). This task involves finding words and phases in text that refer to concepts of interest, and categorising them according to the semantic category that they represent. For example, a medically-relevant NE recognition tool may be expected to recognise *tuberculosis* as an instance of a *disease*, *cold sweats* as an instance of a *symptom*, etc.
- Automatically detecting variants/synonyms of NEs that occur in text (e.g., *scarlatina* as a historically relevant synonym of *scarlet fever*).
- Identifying and classifying relationships involving NEs that occur in text. This includes assigning semantic classes both to the relationships themselves (e.g., *causality*) and to the individual entities involved. The latter type of categorisation helps to differentiate, for example, between cases where tuberculosis plays the role of a *Cause* (e.g. *tuberculosis causes death*) or a *Result* (e.g., *infected milk causes tuberculosis*).

The results of applying such tools to large document archives can allow the development of sophisticated, semantic search systems that provide functionalities such as the following:

- Automatically expanding user-entered query terms with synonyms, variants and other semantically-related terms, in order to aid in the retrieval of a maximal number of potentially relevant documents.
- Using automatically identified semantic information (e.g., NEs and relationships between them) as a means to isolate documents of greatest interest and/or to help users to explore the contents of large result sets from a semantic perspective. Examples include:
  - Restricting results to those in which a search term of interest has been identified as an NE belonging to a specific category (e.g., those documents in which at least one instance of the word *consumption* has been identified as referring specifically to a disease).
  - Exploring the different types of NEs that have been recognised within the result set, as a means of gaining an overview of the scope of information covered within the documents retrieved. For example, after searching for *tuberculosis*, one could view all *drug* NEs that occur within the retrieved documents. This could act as a starting point for discovering the potential range of drugs used in the treatment of tuberculosis.
  - Restricting results to those containing a *relationship* of interest. The high-level semantic representations of relationships that can be produced by TM systems make it possible for users to specify, e.g., that they are looking for documents containing a *Causality* relationship, where *tuberculosis* has been identified as the result. Such a query would allow the location of documents that specifically mention causes of tuberculosis, without the need to enumerate the different ways in which the causality may be expressed within the text. Accordingly, documents will be retrieved in which the relationship may be specified in various different ways, e.g., as an active or passive verb (*X causes tuberculosis* vs. *tuberculosis is caused by X)*, or as a noun (*X is the cause of tuberculosis*).

TM tools usually need to undergo adaptation to make them suitable for application to a given text type or subject area. Important resources needed to support the adaptation process include the following:

- Domain-specific terminological resources, in which concepts are listed, along with their semantically-related terms (e.g., synonyms/variants).
- “Gold standard” annotated corpora, i.e., collections of domain-specific texts in which domain experts have manually marked up various levels of semantic information that are relevant to the domain in question, such as NEs and relationships beween them.

Whilst terminological resources can be used for tasks such as query expansion in search interfaces, annotated corpora are frequently used to *train* tools how to recognise NEs and relationships in the target text type, using *supervised learning techniques*. Such techniques involve applying machine learning (ML) methods to the annotated corpora, in order to try to derive general patterns that encode the characteristics and/or textual contexts of the manually annotated information. For example, the ML process may learn that a noun that is preceded by *suffer from* is likely to correspond to a disease concept. The output of the ML process is a *model* which, using the characteristics and patterns learnt, can be applied to automatically recognise the target semantic information of interest in previously unseen text.

The work described in this article is concerned with adapting TM techniques to the important domain of medical history, which has previously received little attention from a TM viewpoint. Specifically, we are concerned with the development of the necessary resources and tools to facilitate the TM analysis of various types of published documents on medically-related matters, dating back to the mid 19^th^ century. This task presents a number of challenges, according to the variant characteristics that can be exhibited by such documents, which may be subject to evolution as time progresses. These varying characteristics include not only potential shifts in terminology, but also possible variations in writing styles, according to the author, subject matter and intended audience of documents, together with changes in vocabulary and language structure over time. Such characteristics introduce difficulties not only in developing suitable terminological resources, which must account for the various ways in which concepts may be expressed in text both *within* and *across* different time periods, but also in creating annotated corpora that are fit for purpose. Since TM tools developed using ML methods tend to be highly sensitive to the features of the text on which they are trained, an annotated corpus that is suitable for training tools whose aim is recognise semantic information in text with such variant characteristics must include sufficient evidence about the different ways in which the target semantic information may be expressed.

### Related work

Although applying TM techniques to historical medical text is a new area of research, previous work has been carried out on developing TM methods for both modern medical text and historical documents belonging to other subject areas. In the domain of medicine, for example, several annotated corpora have been created [2–7]. Most such corpora consist of modern clinical records, i.e., reports written by doctors about individual patients, which are normally intended only to be read by other doctors. Clinical records are often written in an informal style, which can be very different from the more formal register usually adopted for documents that are to be published, i.e., the types of documents that have been the target of our current research effort. Additionally, we are interested in a rather diverse range of document types. This, combined with the demonstration that TM systems developed for modern text do not necessarily work well on historical text [8], means that modern clinical corpora are unlikely to be useful in our scenario.

Supervised TM methods typically use patterns of linguistic features in learning how to detect the types of semantic information annotated in gold standard corpora automatically, e.g., part-of-speech tags (such as *noun* or *verb*) and syntactic parse results (i.e., structural relations between words and phrases in a sentence, such as a verb, its subject and object). The accurate recognition of such features is often a prerequisite to the accurate extraction of semantic information since, e.g., NEs frequently consist of sequences of nouns and adjectives, whilst NEs involved in relationships usually occur as the subject and object of a relevant verb. To maximise the accuracy of linguistic processing tools when they are applied to different text types, certain such tools have been customised both for specific domains [9, 10] and for historical text processing [11–16]; the output of such tools can in itself help to support search and analysis of historical text collections [17].

Automatic processing of historical text can be affected not only by the different features of the text, compared to modern documents, but also because the only efficient means of making huge volumes of old printed material available in machine-processable format is to carry out scanning of the documents and application of optical character recognition (OCR) procedures. Issues such as poor/variable print quality, or the use of unusual fonts or layouts in the original documents, can contribute to many text recognition errors [18]. Such errors can significantly affect the quality of linguistic processing tools [19], and subsequently the recognition of semantic-level information [20]. A further major issue for historical TM is the scarcity of suitable semantically annotated corpora on which to perform training, given the effort and expense required to create them.

Due to a combination of the above issues, a number of historical TM efforts have either completely or partially abandoned the usual ML-based supervised approach to NE recognition. Instead, the methods employed are either based upon, or incorporate, hand-written rules (which attempt to model the textual patterns that can signify the existence of NEs) and/or dictionaries that contain inventories of known NEs (e.g., [21–26]). Such methods tend to be less successful than ML-based approaches. Firstly, the potentially wide variety of textual contexts, formats and characteristics of NEs means that manually constructed rules are usually less able to generalise than ML models. Secondly, it is difficult to ensure that domain-specific dictionaries provide exhaustive coverage of all concepts, along with their synonyms and variant forms. Nevertheless, there have been a number of efforts to create specialised lexical resources that account for the evolving ways in which concepts are referenced in text over time (e.g., [27–29]).

In terms of identifying relationships between NEs, the difficulty in obtaining accurate syntactic parse results from “noisy” OCR text [30] means that using structural information to aid in the identification of such relationships is not always an option. Instead, identifying co-occurrences (e.g., in the same sentence) between the NEs and/or search terms in historical texts has been demonstrated as an effective means of uncovering important trends and relationships (e.g., [31–33]). In [34], this technique is used to study location-specific changes in the incidence of certain infectious diseases over time.

In order to explore historical medical archives in detail, it is important to take into account the numerous and potentially time-sensitive ways in which diseases and other medically relevant concepts can be referenced in text. As has been explained above, terminological resources have the potential to make searching easier, by providing the means to suggest how queries can be expanded to include variants, synonyms, etc. Indeed, various high-quality, manually curated terminological resources exist for the medical domain, which include variants/synonyms, as well as other types of semantic relationships (e.g., more specific or more general concepts) and which can have very wide-ranging coverage (e.g., [35, 36]). However, they are not designed to provide comprehensive historical coverage, which can make their use problematic in a scenario such as ours, where finding semantic relationships between modern and historical terms is important.

Although many existing terminological resources have been created using manual curation methods, this can be an extremely time consuming task, and large-scale resources can take years to construct and/or update. Accordingly, TM methods are increasingly being explored as a more rapid means to build or augment resources in a (semi-) automatic manner. Techniques include processing text corpora to find new terms that have similar forms to existing dictionary entries [37–39], exploiting textual patterns that reveal relationships between terms [40, 41], extracting structured information contained within specialised historical resources [28], using large-scale Web knowledge bases to increase the coverage of small-scale concept lists derived from historical documents [27] and exploiting the observation that terms that appear in similar textual contexts often exhibit similar meaning [42, 43].

This latter observation is the basis of distributional semantics models (DSMs), which are applied to large text corpora to determine the contextual behaviour of the terms occurring within them. Context may be modelled in various ways, for example, by finding the patterns of words that typically occur before/after a term or by using syntactic information (e.g., finding the set of verbs for which the term can appear as a subject). Terms that are likely to be semantically related are then found by determining those terms whose contexts are very similar to each other. The utility of applying DSMs in automatically generating or augmenting thesauri has been demonstrated (e.g., [44–46]). DSMs present the advantage over some of the approaches introduced above, in that they can be applied to construct new terminological resources without the need for any external knowledge resources apart from a text corpus (although the corpus must be sufficiently large to allow term contexts to be modelled accurately). The nature of DSMs also means that, unlike methods that find related terms based only on lexical-level similarities (i.e., the related terms have similar forms), DSMs can find terms whose forms are completely unrelated, and yet whose meanings are similar (e.g., *smallpox* vs. *variola*). Using information derived from DSMs has been shown to be useful in modelling language behaviour in domain specific text (e.g. [47]), and the utility of such models in processing medically-relevant text has begun to be explored (e.g., [48–50]). In another recent study, applying DSMs to medical corpora containing heterogeneous text types (i.e., both medical journal articles and clinical records) was found to be advantageous in the automatic detection of synonyms [51]. Further relevant work has demonstrated that, when applied to corpora exhibiting temporal variation, DSMs can be exploited successfully to detect evolution in terminology over time [52, 53].

### Research aims

Our work has the following aims:

- To facilitate the application of more sophisticated TM methods to historical medical text than those applied in most related historical efforts.
- To aid in the robust recognition of semantic information of historical and medical relevance in diverse published document types originating from different time periods, from the mid 19^th^ century onwards.
- To provide the means to develop semantic search systems that allow medical historians to search and explore relevant documents in an efficient manner, and to study various aspects of historical change.

**Fig 1:**
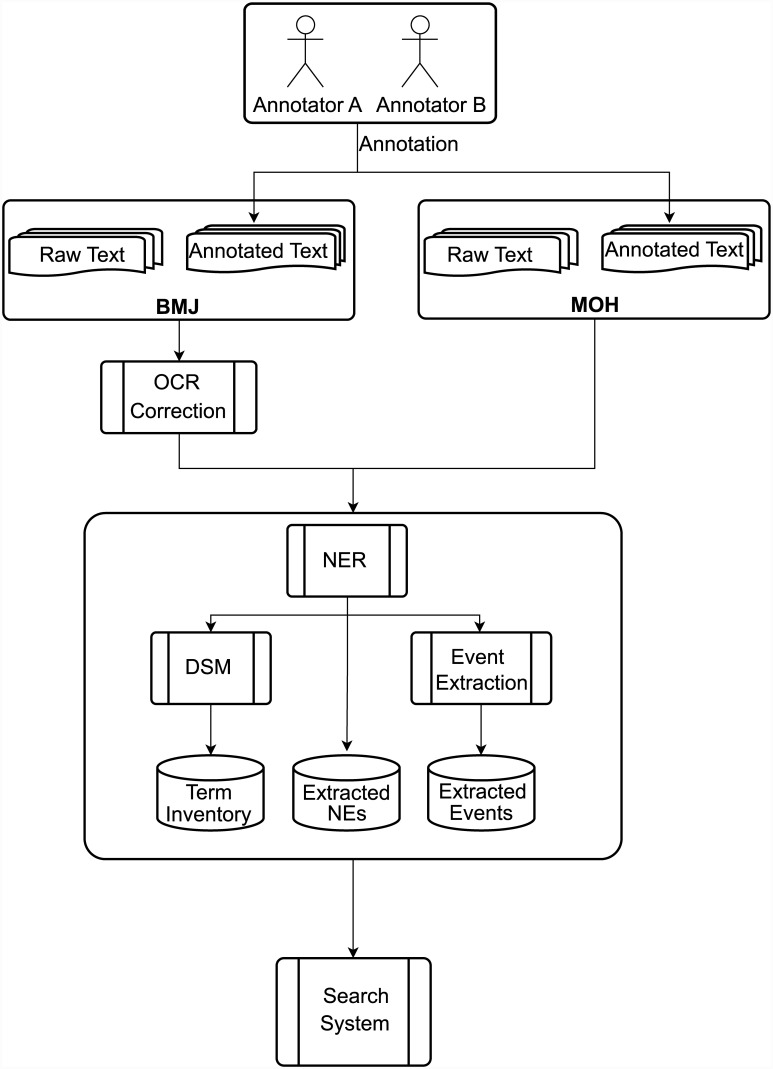
Overview of work.

Fig 1 provides a general overview of all stages of our work, which are described in detail in the subsequent sections of the article. The first stage of our work was to create novel resources, i.e., a terminological inventory and annotated corpus, to support the above aims. We considered it highly important to ensure that these resources take into account the special features of our target area of application, i.e., the potentially large and time-sensitive variations in textual characteristics. This was achieved by drawing upon evidence from two large and varied archives of historical medical text, the British Medical Journal (BMJ) (http://www.bmj.com/archive) and the London Medical Officer of Health reports (MOH) (http://wellcomelibrary.org/moh/), whose documents collectively span the period from 1840 to the present day, and each of which has a different focus (i.e., professional medical matters vs. public health issues). The terminological inventory is available at http://metashare.metanet4u.eu/go2/medical-inventory and the annotated corpus is available at http://metashare.metanet4u.eu/go2/himera-corpus.

We used the annotated corpus to adapt TM tools using ML techniques, and created a modular, interoperable text processing pipeline (or *workflow*) that is tailored to the extraction of semantic information highly relevant to historians of medicine. The ability of this workflow to robustly recognise such information in documents of different types and ages has been demonstrated through its application to the complete contents of the BMJ and MOH archives. The workflow (named *Workflow for History of Medicine*) has been made available via the Argo TM platform (http://argo.nactem.ac.uk/).

The utility of both the semantic data extracted by the workflow and the information contained within our novel terminological inventory has been exemplified through their use in the development of a publicly accessible semantic search interface (http://www.nactem.ac.uk/hom/), whose functionalities include those outlined in the *Background* section above, and which is aimed at allowing medical historians to search and explore information in an efficient manner, and to study various aspects of historical change.

## Methods

In this section, we cover the first part of our work, i.e., the creation of novel resources in support of applying TM methods to 19th and 20th century medical documents. As explained above, the development of these resources was guided through evidence obtained from two large and diverse archives of published historical medical text, each of which represents different points of view on medical matters, and has different target audiences. Brief details of these archives are as follows:

- The BMJ is aimed at medical professionals, and includes various types of articles, including research, analysis, practice, case reports, letters and obituaries. We have worked with an archive consisting of approximately 380,000 articles, spanning from 1840 to 2013.
- The MOH reports are concerned with examining public health issues in different London boroughs. The archive consists of around 5,000 reports produced between 1848 and 1972, whose lengths range from a few pages to several hundred pages.

A subset of the documents in these archives has been used as the basis of development of our annotated corpus, which we have named HIMERA (HIstory of Medicine CoRpus Annotation), and whose creation is described in the first part of this section. The documents in HIMERA, chosen as being representative of the different writing styles and periods covered in the complete archives, have been manually annotated with NEs and their relationships according to our novel annotation scheme. This scheme was designed in conjunction with domain experts to encode various types of semantic information that are relevant from a historical medical perspective. HIMERA provides evidence about how these specific types of information are expressed in heterogeneous document types, with the aim of facilitating the training of ML-based TM tools that can robustly detect NEs and their relationships in published medical documents, regardless of their particular features.

It should be noted that, although previous work has reported on the difficulties in applying ML techniques to certain historical document types, according either to poor OCR quality, which can significantly degrade the output quality of linguistic processing tools, or to the lack of such tools that are suitably adapted to the text type in question, we consider this not to be an issue in our case. Firstly, we found the OCR quality in the MOH archive to be extremely high, due to rigorous, manually-assisted post OCR checks, whilst the OCR text in the BMJ archive, which is sometimes of much lower quality, can be improved significantly through the application of our OCR post-processing method [54]. Secondly, a study into the feasibility of applying modern linguistic processing tools to older texts suggests that their application to 19^th^ century texts should be sufficiently accurate [13], whilst tools specialised for biomedical text have also been shown to work well on medical text [55, 56].

In addition to our use of parts of the BMJ and MOH archives as the basis for the creation of HIMERA, the complete archives have also been employed as a rich source of evidence regarding medical terminology usage and variation over time. Although the UMLS Metathesaurus [35] is a resource that covers vast amounts of medical terminology, we performed experiments revealing that its coverage of historical terminology is severely deficient. Thus, inspired by previous work, which has demonstrated both the advantages of applying DSM methods to heterogeneous textual sources and their feasibility to detect terminology evolution when applied to temporally diverse corpora, we have chosen to apply DSMs to the entire contents of the two archives, in support of the automatic creation of a unique “time-sensitive” terminological resource for medical history. Each term listed in the resource includes synonyms/variants and a range of other semantically related terms. The nature of the archives means that the DSMs have been able to identify semantic relationships between terms that may be synchronic (i.e., the related terms are used within the same time period) or diachronic (i.e., the related terms are used at different periods in time).

### Annotating medical history

Our semantic annotation scheme aims to encapsulate information that occurs commonly within both the BMJ and MOH archives, and which is interesting from a medical history viewpoint. Related efforts to annotate information in clinical records (e.g., [2–7]) are largely focussed on identifying medical disorders, signs, symptoms, treatments and tests, and/or relationships that link them together. Clinical records share some characteristics with a specific type of article in the BMJ, i.e., case reports, in that they are both concerned largely with individual patients, their characteristics, medical history, conditions suffered, investigations undertaken, treatments administered, etc. However, the BMJ as a whole, and the MOH reports, cover a more diverse range of subjects, and examine medical conditions from a broad range of perspectives, which can vary over time. Accordingly, we considered it necessary to develop a customised annotation scheme.

Our novel scheme results from a detailed analysis of a wide range of documents from both the BMJ and MOH archives, which revealed a number of prevalent and recurring themes:

- Discussion of the possible causes of a condition, sometimes by drawing conclusions from a range of cases examined or by carrying out laboratory experiments.
- Identification of the symptoms associated with a condition.
- Identification of the parts of the body that are most typically affected by conditions.
- Discussion of the factors (e.g., therapies, drugs, environmental surroundings) that can affect a condition.
- Discussion of which subsets of the population (e.g., children, adults, ethnic groups, people working in different occupations) are most likely to be affected by a condition.

#### NE types

We have chosen to annotate seven NE types (see Table 1), with the aim of capturing most types of information identified in the recurrent themes above. The definitions of the entity types have been intentionally kept quite broad, so as to minimise the manual annotation burden, i.e., to try to keep the number of decisions that annotators must make to a minimum.

**Table 1:** Annotated NE types.

Entity Type	Description	Examples
**Condition**	Medical condition/ailment	phthisis, bronchitis, typhus
**Sign_or_Symptom**	Altered physical appearance/behaviour as probable result of injury/condition	cough, pain, rise in temperature, swollen
**Anatomical**	Entity forming part of human body, including substances and abnormal alterations to bodily structures	lung, lobe, sputum, fibroid
**Subject**	Individual or group under discussion	children, asthma patients, those with negative reactions to tuberculin
**Therapeutic_or_Investigational**	Treatment/intervention administered to combat condition (including diet/foodstuffs), or substance, medium or procedure used in investigational medical or public health context	atrophine sulphate, generous diet, change of air, lobectomy
**Biological Entity**	Living entity not part of human body, including microorganisms, animals and insects	tubercle bacilli, mould, guinea-pig, flea
**Environmental**	Environmental factor relevant to incidence/prevention/control/treatment of condition. Includes climatic conditions, foodstuffs, infrastructure, household items or occupations whose environmental factors are mentioned	humidity, high mountain climates, infected milk, linen, drains, sewers, dusty occupations.

In contrast to most other related efforts, but similarly to [57, 58], we distinguish between *Condition* and *Sign_or_Symptom*, since our consultations with medical historians revealed the importance of exploring relationships between these two different entity types. We follow other previous work in annotating *Anatomical* locations that are affected by conditions, signs and symptoms, using a similar scope to [59], but without the fine-grained distinctions. The frequent discussion of the medical details/characteristics of different individuals, groups or population sub-types motivates our use of the *Subject* category.

The *Therapeutic_or_Investigational* category encapsulates the diverse range of measures employed in treating, investigating or preventing conditions over the history of the archives, whilst *Biological_Entity* is used to capture the often important mentions of living entities other than the human body, including microorganisms cited as a contributing factor of specific conditions and different animals used in an investigational context. The *Environmental* category is also of particular importance, given the frequent mentions of environmental factors, particularly in older documents and/or those concerned with public health issues.

#### Relationships

Most relationships of interest identified through our manual analysis outlined above are concerned with *causes* and *effects* that involve entities. The prevalence of such relationships in both biomedical articles and clinical text has previously led to several efforts to annotate and recognise them automatically at several levels of granularity (e.g., [60–62]).

**Fig 2:**
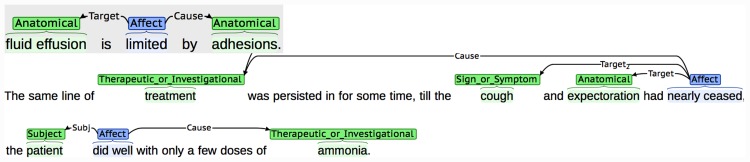
Examples of annotated *Affect* events. Event triggers are shown in blue and entity annotations are shown in green. Event participants are linked to the corresponding event trigger with arrows. The labels on the arrows represent the semantic label assigned to the participant.

**Fig 3:**
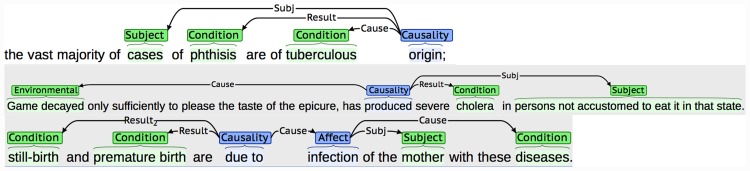
Examples of annotated *Causality* events. Event triggers are shown in blue and entity annotations are shown in green. Event participants are linked to the corresponding event trigger with arrows. The labels on the arrows represent the semantic label assigned to the participant.

We have chosen to annotate relationships as specific types of semantic structures called *events*. Events consist of two broad types of text span annotations, which are linked together in different ways to encode potentially complex interactions between entities and/or other events. Firstly, each event has a single *trigger* (a word, typically a verb or verb phrase, that characterises the nature of the relationship). This is linked to one or more of the second class of text span annotations involved in events, which are called *participants*. Participants correspond to entities or other events that contribute towards the description of the relationship. Both complete events and their participants are categorised through the assignment of different types of semantic labels, some examples of which are provided in Table 2. The use of event structures is advantageous not only in terms of their ability to capture complex relationships, but also since automatic event recognition is supported by various state-of-the-art ML-based tools (e.g., [63–65]). Table 2 provides a brief description of the two types of events that we have defined, while Figs 2 and 3 illustrate some specific examples from the BMJ and MOH archives.

**Table 2:** Annotated event types.

Event Type	Description	Possible Participants
**Affect**	A (previously existing) entity or event is affected, infected, undergoes change or is transformed, possibly by another entity or event.	**a) Cause:** Cause of the affection; **b) Target:** Entity or event affected; **c) Subj:** Individual or group affected
**Causality**	An entity or event results in the manifestation of a (previously non-existing) entity or event.	**a) Cause:** Cause of the manifestation; **b) Result:** New entity or event that manifests itself; **c) Subj:** Individual or group associated with the event

### HIMERA corpus composition

The composition of the HIMERA corpus aims to reflect the diversity of document types, writing styles and time periods covered by the archives, so as to allow the training of ML models that are robust to such variations. Medical history experts used the following document selection criteria:

- Documents should mostly cover lung diseases. This choice arose from consultation with our advisory board, drawn from the history of medicine community. Various options were explored, keeping in mind the relatively short duration of our project, and priority was assigned to lung diseases, due to their significance for study over long periods of time. HIMERA is thus especially focussed on, but not restricted to, facilitating the training of systems able to recognise information surrounding this class of disease.
- A range of documents should be chosen from both the BMJ and MOH archives.
  - Given the large size of many MOH reports, relevant *extracts* should be selected from complete reports.
  - BMJ articles should be representative of the variety of article types, including letters, case reports, leading articles, etc.
- Given our focus on documents of a more historical nature, and to account for potential time-sensitive differences/changes, texts should be chosen from four key decades in medical history: 1850s, 1890s, 1920s and 1960s

For each document in HIMERA, OCR errors were hand-corrected by the experts with reference to the original scanned pages, in order to create high-quality, gold standard text on which to perform annotation. The complete HIMERA corpus (around 70,000 words) includes 35 BMJ articles (evenly distributed amongst the four decades) and excerpts from four MOH reports (one for each decade).

#### Annotation procedure

Manual annotation was performed by different annotators using *brat* [66], a Web-based tool that is specifically designed for rapid annotation of textual documents. It is easily configurable to new annotation tasks and has support for both NE and event annotation. To reduce annotation burden, we carried out automatic pre-annotation of certain NE types; the automatically added annotations were subsequently reviewed, deleted or augmented by annotators. As the basis for the pre-annotation, we applied a tool called MetaMap [39] to the documents of the HIMERA corpus. MetaMap is able to recognise a wide range of medical NE types and, although aimed at modern text, recognises a number of types that correspond closely to (subsets of) our chosen entity categories. We thus defined various mappings between MetaMap and HIMERA entity categories as shown in Table 3. In some cases, filtering rules/restrictions were used to increase the accuracy of the mapping.

**Table 3:** MetaMap to HIMERA category mappings.

MetaMap Categories	HIMERA Category	Filtering Rules
Anatomical Abnormality, Body Substance, Body Part, Organ, Organ Component, Body Location or Region, Body Space or Junction, Tissue	Anatomical	
Animal, Mammal, Cell, Bacterium, Organism	Biological_Entity	
Food, Chemical Viewed Structurally, Element, Ion, or Isotope, Hazardous or Poisonous Substance, Substance, Natural Phenomenon or Process	Environmental	
Disease or Syndrome, Pathologic Function	Condition	Must be a noun phrase
Clinical Drug, Amino Acid, Peptide, or Protein, Immunologic Factor, Organic Chemical, Pharmacologic Substance, Biologically Active Substance, Lipid	Therapeutic_or_Investigational	Must be a noun phrase; Length must be greater than 1
Sign or Symptom, Finding	Sign_or_Symptom	Tagged as noun
Group, Patient or Disabled Group	Subject	

To ensure that the annotated corpus can be used to train accurate ML models, it is important that different human annotators should produce consistent annotations. To verify that annotators have a common understanding of the scheme and how it should be applied, it is usual for a portion of the corpus to be annotated independently by each annotator. Inter-annotator agreement rates can then be calculated, to determine the level of consistency between the annotations produced. In our case, approximately a quarter of the corpus was double-annotated for this purpose; inter-annotator agreement rates for entities are shown in Table 4. In common with several other related efforts (e.g., [67, 68]), agreement rates are reported in terms of F-Score (an evaluation measure originally introduced for information retrieval systems), for both *exact* matches, where the start and end of the annotated text spans chosen by both annotators match exactly, and *relaxed* matches, where it is sufficient for the annotations to include some common parts.

**Table 4:** Entity annotation agreement rates.

Type	Agreement rate (F-score)	
	Exact Match	Relaxed Match
**Anatomical**	0.81	0.85
**Biological_Entity**	0.99	0.99
**Condition**	0.92	0.95
**Environmental**	0.63	0.79
**Sign_or_Symptom**	0.84	0.88
**Subject**	0.70	0.81
**Therapeutic_or_Investigational**	0.73	0.78
**TOTAL**	0.80	0.86

Table 4 illustrates that high levels of inter-annotator agreement are achieved for most categories. There are generally quite small differences between exact and relaxed matching rates, showing that annotators are normally clear about the exact span of text to annotate. Categories with wider semantic scope (e.g., *Environmental* and *Therapeutic_or_Investigational*) have slightly lower agreement rates, whilst differences between exact and relaxed matching rates are greatest for *Subject* and *Environmental*, whose variable lengths and/or internal structures can make it more difficult to decide on the exact span of text to annotate. Although annotations for both of these categories most commonly correspond to short noun phrases, a more complex phrase is sometimes needed to accurately characterise the entity, e.g., *those with negative reactions to tuberculin*, *patients surviving five years or more*, *free circulation of air*.

Table 5 shows the number of entities annotated in the complete HIMERA corpus. To ensure the highest possible quality of the annotations, a senior medical historian was responsible for reviewing all annotations, in order to create the final gold standard corpus. This activity involved adjudicating discrepancies in double annotated documents and reviewing/editing the annotations in single annotated documents. There is a fairly even distribution of most entity types, although *Anatomical* entities are the most frequent. This can be explained by their occurrence in various contexts, e.g., in the descriptions of the locations/characteristics of *Condition* and *Sign_or_Symptom* entities, and to refer to abnormal structures, such as tumours or tubercle. In contrast, *Biological_Entity* instances are relatively uncommon, partly due to the rare mention of microorganisms before the 20th century, and also since mentions of mammals, insects, etc., are normally peripheral to the main focus of documents.

**Table 5:** Annotated Entity counts in HIMERA.

Type	Count
**Anatomical**	2002
**Biological_Entity**	295
**Condition**	1499
**Environmental**	1268
**Sign_or_Symptom**	1171
**Subject**	1062
**Therapeutic_or_Investigational**	1046
**TOTAL**	8343

### Time-sensitive inventory of historical medical terms

To support assisted, historically-aware query expansion in search interfaces, it is necessary to have access to a domain-specific terminological resource that includes synonyms/variants that occur both synchronically and diachronically, as well as other terms with close semantic links.

The UMLS Metathesaurus [35] covers a huge number of medical concepts, including variant and synonymous terms, and has been shown to be useful for query expansion purposes (e.g., [69–71]). However, its coverage of historically relevant terminology, and hence its utility in our scenario, was initially unclear. To evaluate this, we processed the OCR text from two 19^th^ century disease terminologies [72, 73] from the National Library of Medicine’s Digital collections (http://collections.nlm.nih.gov/), each of which lists synonyms for each concept. Exploiting the regular structure of these resources, we extracted sets of synonymous terms, each being representative of a particular historical disease concept. We then tried to link as many as possible of these historical concepts with UMLS concepts. A link was established if at least one of the terms associated with a historical concept either matched or closely resembled a term associated with a UMLS concept.

In this way, we were able to link 1,588 historical concepts to modern concepts, but we found that 2,422 historical *terms* did not have an equivalent in the UMLS Metathesaurus. For example, the term *epidemic meningitis* appears in both the UMLS Metathesaurus and one of the historical resources, allowing a link to be established between the concepts in the two resources. However, the term *cerebro-spinal fever* only occurs as a synonym of *epidemic meningitis* in the historical resource and not in the UMLS Metathesaurus. The above findings, which consider only diseases, suggest that there are many historically relevant variants of UMLS concepts that are not listed in the Metathesaurus. We also found that over 800 historical terms could not be linked to any UMLS concept, suggesting either that there are entire historical concepts that are no longer relevant, or else that there is no overlap between the terms that were used to describe a concept in the past and the terms that are used today.

Although historical terminological resources clearly contain useful information about terminology usage in the past, the employment of such resources as a complete solution for acquiring historical terminology can be problematic: each resource generally has a different format and thus requires ad-hoc processing to extract the required information automatically. Additionally, terms listed in these manually curated resources may not fully account for all variants that are actually used in text.

#### Distributional semantic models

According to the issues highlighted above, we have complemented the extraction of terms from historical terminology resources with the results of applying DSMs to our archives. This has allowed the discovery of a greater range of semantically related terms from different time periods, and which, importantly for TM purposes, are based on evidence of real usage in text.

DSMs aim to discover, for each input term (source), a set of semantically related terms (targets), based upon similarities in their textual contexts in a large corpus of texts. We applied the following steps to the contents of the BMJ and MOH archives:

1. The range of possible contexts (or *context distributions*) for a set of source terms was determined, using different models, as explained below. The context of each source term is represented using the base forms (lemmas) of words, (e.g., singular forms of nouns, infinitives of verbs), that can occur within a window of three words before and three words after the term in the two archives. Lemmas were obtained by pre-processing the documents in the archives with a linguistic processing tool, the TreeTagger [74]. A set of *stop-words* (e.g., *the*, *a*, *of*, *to*) was excluded from the context distributions, to try to ensure that only “meaningful” words were used as contextual evidence. We refer to these remaining lemmas as *lexical units*.
2. A measure of similarity (*cosine similarity*) between the context distributions of source and target terms was computed. For each source term, the 20 target terms with contexts bearing the greatest similarity to the source term were retained.

The standard approach (SA) to compiling context distributions is the count-based model. We collected the 150,000 most frequently occurring lexical units in the archives. For each source term, we determined all such lexical units that occur within the specified window. For each lexical unit that occurs in the context of a source term, a value (the *log-likelihood ratio*) is assigned to denote its degree of correlation with the source term. The set of lemmas, accompanied by their log-likelihood values, constitutes the context distribution or *context vector* for the source term.

Whilst the SA approach can work well for single word terms, compiling sufficiently detailed and accurate context distributions for multi-word terms can be a much greater challenge; multi-word terms normally occur much less frequently than single word terms, meaning that less information will be available about their contextual distributions. Accordingly, alternative methods have been proposed, which use different means that attempt to *approximate* information about the contextual distribution of multi-word terms. Compositional DSMs exploit the *principle of compositionality*, which states that the meaning of a multi-word phrase is often represented as a function of the meaning its constituent words [75]. Thus, instead of calculating contextual distributions of multi-word terms based only on instances of their *complete* occurrences in text, compositional DSMs combine the context vectors of the individual words that make up a multi-word term in various ways, in order to estimate the likely contextual distributions of multi-word terms. Compositional DSMs thus assume that, since the individual words in a multi-word term contribute towards the overall meaning of the term, there will be some degree of shared context between occurrences of the component words and occurrences of the complete multi-word term.

Our experiments use two basic methods of compiling compositional DSMs, as introduced in [75]. The Basic Additive Model (BAM) simply combines the context vectors for all constituent words in the multi-word term into one large vector. By using this method, all lexical units that occur in the context of any of the constituent words of the term are considered to constitute potential context for the complete multi-word term. Although this method can build up large and detailed context vectors for multi-word terms, such vectors are not necessarily completely accurate–it may be the case that some multi-word terms share very little context with that of their constituent words. To try to alleviate such potential problems, the Basic Multiplicative Model (BMM) takes a different approach, by calculating the element-wise product of the context vectors of the constituent words of the multi-word term. Using this method, only those lexical units that are shared between the contexts of *all* constituent words of the multi-word term are considered to constitute relevant context for the complete term; considering only such shared contexts means that BMM is able to better take into account the potential interactions between the component words of the term and is thus more likely to produce a context vector that more accurately models the contexts in which the whole multi-word term appears.

The drawback of BMM is that it can require very large corpora to obtain an accurate representation of the shared contexts of all component words appearing in all multi-word terms; in small corpora, some component words may not occur very frequently, meaning that the resulting context vector for the multi-word may be skewed. In contrast, BAM is less affected by the size of the available corpus. Even if one or more of the component words of a multi-word occurs only rarely in the corpus, then the fact that BAM includes the context vectors for all other component words without restriction means that a variety of potential contexts for the multi-word term will still be included within the context vector.

In our work, we have applied all of the three models introduced above, i.e., SA, BAM and BMM, to the BMJ and MOH archives; the results are discussed below in the *Results* section.

## Results and Discussion

In this section, we firstly explain how we used the HIMERA corpus to train domain-specific NE and event recognition tools that are robust to stylistic and temporal variations; we have incorporated these tools into our interoperable TM pipeline for enriching historical medical text with semantic information. We subsequently report on the evaluation of the DSMs when applied to the BMJ and MOH corpora, in order to determine the most suitable model to apply in the automatic creation of our complete temporal inventory of historical medical terminology. We evaluate these models in two ways: firstly, we carry out a quantitative evaluation, by determining the extent to which the models can automatically detect synonyms/variants that are already listed within the UMLS Metathesaurus. This helps to provide a general indication of the relative abilities of the different models to recognise synonyms in our chosen data sets. However, given that the whole point of applying DSMs is to recognise semantically related terms that are *not* present within the UMLS Metathesaurus, we supplement our quantitative evaluation with an expert qualitative evaluation, whose aim is to determine the extent to which historically relevant synonyms/variants, as well as other types of semantically related terms, can be detected using DSMs.

### NE recognition

We have trained our NE recognition tool by applying an existing software package to the annotated HIMERA corpus. The package, called NERSuite (http://nersuite.nlplab.org/), is specifically designed to facilitate the development of ML-based NE recognition tools. Given the previously mentioned importance of various types of linguistic information in the accurate prediction of semantic information, NERSuite comes with built-in functionality to carry out linguistic pre-processing, using the biomedically-tuned GENIA tagger [9]. The suitability of NERSuite for application in our scenario is reinforced by its previous use in the development of a number of other medically-relevant NE tools, which achieved high levels of performance [59, 76].

The linguistic features obtained through the application of the GENIA tagger, and which are used as input to the learning process, consist of the surface and base forms of words, part-of-speech tags and syntactic chunks (e.g., a noun phrase chunk may consist of a determiner, such as *the* or *a*, adjectives and nouns). However, it is possible to augment these linguistic features with semantic features, e.g., concept categories, which can be assigned to words and phrases by looking them up in domain-specific terminological resources, such as the UMLS Metathesaurus. Such features can help, for example, to ensure that the NE recognition tool can correctly detect instances of NEs that are already listed within these resources and/or new NEs in which part of the complete span corresponds to a known NE. The use of semantic features has been demonstrated to improve performance in the previously reported NERSuite models relevant to our subject domain, compared to the use of linguistic features alone [59, 76].

We have employed NERSuite to train several models using HIMERA, both with and without semantic features. We carried out two sets of experiments, the first of which was aimed at determining the extent to which a single model can recognise NEs accurately and robustly when applied to texts that originate from different time periods and sources. The second set of experiments had the goal of investigating the extent to which accurate NE recognition is influenced by the time-sensitive nature of our data.

#### Single NE recognition model

To determine the best single NER model, aimed at robust application across all documents of both the BMJ and MOH archives, we carried out experiments with four different sets of features, as follows:

- **Baseline (BL)**–Default set of linguistic features used by NERSuite.
- **Full MetaMap (FM)**–Basic linguistic features are augmented with semantic features from default application of MetaMap (133 semantic types).
- **Selective MetaMap (SM)**–Equivalent to the pre-annotation for the HIMERA corpus, described in the *Methods* section, i.e., selected MetaMap categories (as shown in Table 3) are mapped to our own seven entity categories, under certain conditions.
- **UMLS Lookup (UL)**–Dictionary lookup of word/phrases is performed on a filtered version of the full UMLS Metathesaurus dictionary. The Metathesaurus uses the same semantic types as MetaMap; we retained only those entries corresponding to categories shown in Table 3, and mapped them to our own entity categories.

Although MetaMap is based largely on lookup in the UMLS Metathesaurus, it can potentially recognise a wider and more accurate range of entities than simple lookup in this resource, given that it employs a range of heuristics to recognise concept variants that are not necessarily listed in the Metathesaurus. However, it tends to be rather slow. This has motivated our use of the different experimental settings outlined above, which allow us to evaluate whether there are any significant performance differences between the generation of semantic features using MetaMap and simple lookup in the UMLS Metathesaurus. The latter is considerably quicker, especially since NERSuite incorporates an efficient dictionary lookup mechanism.

Given the relatively small size of HIMERA, our experiments were carried out using 5-fold cross validation. Using this method, the corpus is split into five roughly equal parts (folds). Each fold includes a mixture of documents from different decades and from both archives. The expert-added, gold standard semantic annotations are augmented with (relevant choices of) features discussed above. Five different models are then trained, using different combinations of four out of the five folds. In each case, the fifth fold (the test set) is copied, with the copy being stripped of its gold standard NE annotations and both original and copy of the test set left out of the training data, to be later used for the evaluation of the trained model. For evaluation, each trained model is applied to recognise NEs in the stripped documents of the test set, after which these automatically recognised NEs are compared against the original gold standard annotations for that test set. The final results are calculated by averaging the results obtained from the evaluation of the five different models. Although seemingly complex, cross-validation is a necessary step to avoid potential bias in reporting the results of an ML model, by making sure that the results reported are not over-fitted to a particular data set.

The results obtained using the different feature sets and different matching criteria (i.e., exact and relaxed span matching) are reported in Table 6. The results are reported in terms of precision (the proportion of NEs predicted by the model that are actually correct), recall (the proportion of gold standard NEs that were actually recognised by the model) and F-Score (the harmonic mean of precision and recall, providing a single, generalised measure of performance of the model).

**Table 6:** 5-fold cross-validation NE results.

	Exact Span Match				Relaxed Span Match			
Category	BL-Ex	SM-Ex	FM-Ex	UL-Ex	BL-Rel	SM-Rel	FM-Rel	UL-Rel
**Environmental (P)**	**0.66**	0.63	0.64	0.65	**0.79**	0.76	0.77	**0.79**
**Environmental (R)**	0.39	**0.41**	**0.41**	0.40	0.46	**0.50**	0.49	0.48
**Environmental (F)**	0.49	**0.50**	**0.50**	0.49	0.58	**0.60**	**0.60**	**0.60**
**Condition (P)**	0.78	0.80	**0.81**	**0.81**	0.88	0.89	**0.90**	0.89
**Condition (R)**	0.68	**0.75**	0.74	0.74	0.76	**0.83**	0.82	0.82
**Condition (F)**	0.73	**0.78**	**0.78**	0.77	0.82	**0.86**	0.85	**0.86**
**Subject (P)**	0.70	0.79	**0.80**	**0.80**	**0.87**	**0.87**	**0.87**	**0.87**
**Subject (R)**	0.70	0.70	**0.71**	0.70	0.76	0.76	**0.77**	0.76
**Subject (F)**	0.74	0.74	**0.75**	**0.75**	0.81	0.81	**0.82**	0.81
**Sign_or_Symptom (P)**	0.79	0.79	**0.81**	**0.81**	0.86	0.86	**0.87**	**0.87**
**Sign_or_Symptom (R)**	0.56	**0.62**	0.61	0.60	0.61	0.66	**0.65**	**0.65**
**Sign_or_Symptom (F)**	0.66	**0.70**	**0.70**	0.69	0.71	**0.75**	**0.75**	0.74
**Anatomical (P)**	0.80	0.80	**0.82**	0.80	0.88	0.88	**0.89**	0.88
**Anatomical (R)**	0.66	0.70	0.70	**0.72**	0.72	0.77	0.76	**0.78**
**Anatomical (F)**	0.73	0.75	**0.76**	**0.76**	0.79	0.82	0.82	**0.83**
**Therapeutic_or_Inv (P)**	0.71	0.73	0.72	**0.74**	0.79	0.79	0.80	**0.81**
**Therapeutic_or_Inv (R)**	0.48	**0.52**	**0.52**	**0.52**	0.53	**0.57**	**0.57**	**0.57**
**Therapeutic_or_Inv (F)**	0.57	**0.61**	0.60	**0.61**	0.64	0.66	**0.67**	**0.67**
**Biological_Entity (P)**	**0.91**	**0.91**	**0.91**	**0.91**	0.93	**0.94**	0.93	0.93
**Biological_Entity (R)**	0.57	0.57	0.61	**0.62**	0.58	**0.66**	0.62	0.63
**Biological_Entity (F)**	0.70	0.70	0.73	**0.74**	0.72	**0.78**	0.74	0.75
**TOTAL (P)**	0.77	0.77	**0.79**	0.78	0.86	0.85	**0.86**	**0.86**
**TOTAL (R)**	0.58	**0.63**	**0.63**	**0.63**	0.65	**0.70**	0.69	**0.70**
**TOTAL (F)**	0.67	**0.70**	**0.70**	**0.70**	0.74	**0.77**	**0.77**	**0.77**

BL = Baseline; SM = Selective MetaMap; FM = Full MetaMap; UL = UMLS Lookup; Ex = Exact span matching; Rel = Relaxed span matching; P = Precision; R = Recall; F = F-Score. The best Precision, Recall and F-Score results for each category are shown in bold type.

Since the trained models tend to favour the prediction of shorter spans, there is a certain degree of discrepancy between exact and relaxed span match results, especially for categories more liable to variation in terms of the annotated span length or internal structure. For example, the trained model does not always correctly identify the complete span of NEs that include prepositional phrases at the end, such as *diseases of the respiratory system*. However, recognition of NEs that include adjectives preceding the noun, as in *muscular weakness* or *medical treatment*, is less of a problem for the model.

Precision is high for most categories, i.e., entities predicted by the model are usually correct. Using semantic information normally boosts recall, i.e., it ensures that a greater number of correct entities are recognised by the model, although the amount of recall boost obtained varies for different categories, and is likely to be dependent on factors such as the extent of alignment between the semantics of UMLS categories and our own categories, together with the amount of time-sensitive variation that occurs within a category.

Another important result is that the three models that use semantic information all behave with comparable accuracy. Due to the slowness of MetaMap, the possibility of using basic UMLS dictionary lookup to generate semantic features, with little, if any, loss of model accuracy, is a huge advantage when applying the model to the complete archives. For most categories, an overall performance of around 0.75 F-score or higher (relaxed matching) is achieved when using semantic features. *Environmental* and *Therapeutic_or_Investigational* exhibit somewhat lower recall than other categories, which is likely to be due to their comparatively wider semantic scope.

A further positive outcome of our experiments is that our results compare highly favourably to those achieved by other relevant tools that have employed NERSuite for training. In [77], a relaxed match F-score of 0.78 for anatomical entities is achieved, using a similar experimental setup to ours; a higher relaxed match F-score (0.91) is reported in [59] for the same task, but using a much larger annotated corpus and more complex set of features. F-Scores of 0.88/0.75 (relaxed/exact matches) are achieved by [76] in the recognition of disorders (i.e., diseases or symptoms) in electronic health records as part of the SemEval 2014 shared task [78]; the system was ranked second out of 21 participating systems. The fact that our model achieves similar results for our broadly comparable *Condition* category serves to demonstrate that the performance of our models is on par with state-of-the-art efforts.

#### Time-sensitive NE recognition models

To investigate the impact of the temporal variations in text on NE recognition, our second set of experiments involved splitting the HIMERA corpus according to decades. Taking the documents from each decade in turn as the test set, we evaluated the ability of models trained on one or more decades to correctly predict the entities in the test set (i.e., documents from a different decade to those in the training set). Training was carried out using the UL feature set described above, based upon its favourable results and ease of application in the first set of experiments. The results of these time-sensitive experiments are shown in Table 7.

**Table 7:** Temporal-based model results.

Test data	Training data	Env.	Cond.	Subj.	SS	Anat.	TI	Biol.	ALL
N/A	UL (5-fold)	**0.60**	**0.86**	**0.81**	**0.74**	**0.83**	**0.67**	**0.75**	**0.77**
**1850s**	1890s	0.19	0.80	0.74	0.65	**0.78**	0.28	0.41	0.65
**1850s**	1920s	0.34	0.73	0.70	0.54	0.55	0.37	0.33	0.55
**1850s**	1960s	0.33	0.70	0.61	0.61	0.42	0.08	0.00	0.49
**1850s**	1890s/1920s	0.33	**0.81**	**0.77**	0.67	0.77	**0.4**	**0.44**	0.67
**1850s**	1920s/1960s	**0.47**	0.72	0.73	0.56	0.61	0.34	0.34	0.58
**1850s**	All other dec.	**0.47**	**0.81**	**0.77**	**0.69**	**0.78**	0.37	0.43	**0.69**
**1890s**	1850s	0.32	0.79	0.79	0.68	0.78	0.35	0.41	0.70
**1890s**	1920s	0.26	0.77	0.73	0.56	0.66	0.39	0.87	0.64
**1890s**	1960s	0.23	0.72	0.68	0.41	0.54	0.19	0.13	0.51
**1890s**	1850s/1920s	0.38	**0.85**	**0.80**	**0.72**	0.83	0.47	0.88	0.76
**1890s**	1920s/1960s	0.29	0.67	0.72	0.54	0.66	0.33	**0.92**	0.61
**1890s**	All other dec.	**0.44**	**0.85**	0.78	**0.72**	**0.84**	**0.52**	0.91	**0.77**
**1920s**	1850s	0.37	0.77	0.80	0.61	0.48	0.32	0.22	0.53
**1920s**	1890s	0.17	0.76	0.77	0.64	0.49	0.35	0.66	0.52
**1920s**	1960s	0.15	0.67	0.67	0.28	0.41	0.25	0.10	0.42
**1920s**	1850s/1890s	**0.39**	0.78	**0.81**	0.67	0.54	0.44	0.68	0.59
**1920s**	1890s/1960s	0.28	**0.79**	0.78	0.65	0.55	0.45	**0.75**	0.57
**1920s**	All other dec.	**0.39**	**0.79**	**0.81**	**0.70**	**0.56**	**0.54**	0.74	**0.61**
**1960s**	1850s	0.32	0.77	0.48	0.45	0.47	0.18	0.03	0.47
**1960s**	1890s	0.07	0.74	0.51	0.47	0.57	0.27	0.08	0.46
**1960s**	1920s	0.18	**0.79**	0.44	0.43	0.39	0.37	0.09	0.46
**1960s**	1850s/1890s	0.30	0.78	**0.52**	**0.51**	0.55	0.32	0.07	0.50
**1960s**	1890s/1920s	0.19	0.78	0.51	0.50	**0.62**	**0.50**	**0.16**	0.53
**1960s**	All other dec.	**0.35**	**0.79**	0.51	**0.51**	0.61	0.46	**0.16**	**0.54**

Results are shown in terms of F-Score (relaxed span matching). Relaxed match F-Score UL results from the 5-fold cross validation experiments are shown on the first line, for comparison purposes. For each decade of test data, the bold figures indicate the best performing model(s) for each category of NEs. Env. = Environmental, Cond. = Condition, Subj. = Subject, SS = Sign_or_Symptom, Anat. = Anatomical, TI = Therapeutic_or_Investigational, Biol. = Biological_Entity.

The results shown in Table 7 provide strong evidence that the recognition of certain NE types is dependent upon temporally sensitive features of text, based on the significant performance differences according to the decade(s) of the documents used for training. When models are trained only on a single decade, the use of documents from a neighbouring (usually previous) decade is usually most effective; training on documents from more distant decades generally produces poorer results. When two decades are used for training, it is usually also the case that the use of data from periods neighbouring the test decade tends to produce the best results. A likely explanation is the rapid and incremental nature of medical developments; very old knowledge is likely to become irrelevant, but knowledge introduced in one decade may still be relevant in the next.

Our results also suggest that the availability of training data that is temporally close to the test data period appears to be more important than the *volume* of training data. When three decades are used for training (i.e., *All other dec*. in Table 7), there is usually little, if any, improvement in performance compared to when only the two neighbouring decades are used. Even though the use of all other decades as training data constitutes a similar amount of training data to that used for the original UL 5-fold model, performance is generally lower, presumably since, in this second set of experiments, evidence from the test decade does not figure at all within the training data.

The *Environmental* and *Biological_Entity* categories seem to be less influenced by time; the sparsity of annotations belonging to the latter category may help to explain its unpredictable behaviour, whilst for the former, the quantity of training data often appears to be more relevant than temporally related data.

Our results illustrate the general importance of including data relevant to period of interest within the training data. However, the additional inclusion of data from other periods does not seem to be harmful. This helps to reinforce the suitability of training a single model for application to the entire archives. However, since our results suggest that there can be extreme variation in the means of expression and/or the contexts of certain NE types over time, the accuracy of our current single NE recognition model is likely to benefit from our on-going efforts to expand the HIMERA corpus to include data from additional decades.

### Event recognition

Event recognition is undertaken by our EventMine system [63]. Regular improvements to the system have assured its state-of the-art performance when applied to texts of different types and belonging to different subject areas [79–81]. It has also been shown to perform robustly on very large text collections. EventMine works by applying a pipeline of TM tools to text in which NEs have already been recognised. The different tools are used to recognise event triggers and participants, to assign appropriate semantic categories to them and to link them together into potentially complex event structures. In a similar way to NERSuite, EventMine is ML-based, and is reliant on the output of linguistic processing tools to identify a range of features that are used to aid in the accurate recognition of various parts of the event structure. Whilst some of the features used are similar to those used by NERSuite (e.g., part-of-speech, base form), others are more complex and make use of structural (i.e., syntactic) information. Such features can be particularly important for the accurate recognition of event participants, given that they are frequently structurally linked to event triggers (e.g., they constitute the grammatical subject or object).

We configured EventMine to recognise our *Causality* and *Affect* event types, and its performance was evaluated using 5-fold cross validation, with the same folds as for NE recognition. Table 8 shows the performance of the recognition of event triggers.

**Table 8:** Event trigger recognition results.

Event Type	Exact Match	Relaxed Match
**Affect (P)**	0.41	0.46
**Affect (R)**	0.39	0.44
**Affect (F)**	0.40	0.45
**Causality (P)**	0.36	0.52
**Causality (R)**	0.13	0.18
**Causality (F)**	0.19	0.27
**TOTAL (P)**	0.40	0.47
**TOTAL (R)**	0.32	0.37
**TOTAL (F)**	0.35	0.42

P = Precision; R = Recall; F = F-Score

Whilst the results are considerably lower than for entity recognition, this can be at least partly explained by the small number of events that were annotated in HIMERA (205 *Causality* events and 611 *Affect* events). Thus, compared to NEs, there is relatively little evidence about the characteristics and contexts in which such events can occur, which can be extremely problematic for supervised ML techniques.

It should also be taken into account that the accuracy of syntactic parse results, which are vital for accurate event recognition, can be variable, according to the wide variety of writing styles (and hence language structure) encountered in the archive.

Despite these issues, precision rates for *Causality* events (relaxed matching) seem particularly promising. For *Affect* events, although there is a greater number of training instances than for *Causality* events, the precision for exact matches is only slightly higher than for *Causality* events. A likely reason is that, since the *Affect* event type has a relatively wider semantic scope, which covers affection, infection, transformation and change (e.g., improvement or decline), there is insufficient evidence for each of these sub-types in the training data to allow accurate predictions to be made. In contrast to *Causality* events, however, the larger amount of training data available means that recall for *Affect* events is higher, with a fairly equal balance between precision and recall.

On biomedical text, EventMine can achieve an overall event recognition performance of around 0.57 F-score, which is state-of-the-art. However, such results have only been achieved using much larger amounts of training data than are available in HIMERA. Performance for individual biomedical event types can be much lower, however, when the available training data is as sparse as in HIMERA. It is also the case that biomedical event types normally have much narrower semantic scope than our rather general event types, which can help to make the recognition of the former an easier problem. Finally, the modern academic text that has been used to train existing biomedical event extraction systems is likely to include less stylistic and language variation than is present in HIMERA.

Although our current evaluation does not consider event participants, the results of applying our event model to the complete archives shows that it *is* capable of recognising them. In an attempt to boost current event extraction performance, we are currently working to triple the size of HIMERA, with considerable effort having been made to select documents that contain a large number of relevant events.

### Distributional semantic models

Our research into DSMs has aimed to evaluate the best model to use in the automatic generation of our time-sensitive terminological inventory. Whilst our final term inventory provides terms related to all categories of NEs that can be detected by our NE recognition tool, our evaluation of DSMs was carried out concurrently with our production of HIMERA. Thus, for this evaluation, our historically-oriented NE model was not available as a means of generating suitable source terms for input to the DSMs. Accordingly, we chose disease terms to be the focus of our evaluation, since we could exploit existing resources to generate a suitable set of source terms, and also since they correspond to entities of interest to us (i.e., they constitute a subset of our *Condition* entity category). Specifically, we applied two NE recognisers to our archives: an NERSuite model trained using the NCBI annotated disease corpus of modern biomedical text [82], and a dictionary-based recogniser, based on the terms extracted from the 19th century historical disease terminologies (as described in the Methods section), in order to better account for historically-relevant disease mentions.

#### UMLS-driven evaluation

We firstly evaluated the target terms produced for each source term by the different DSM models, through comparison of the target terms to the synonyms listed for the source term in the UMLS Metathesaurus. For the purposes of the evaluation, we selected as source terms the 500 most common single and multi-word terms that occur more than 20 and less than 1000 times in the archives, and which appear in the UMLS Metathesaurus. We constructed context vectors for each source term, and applied the SA, BAM and BMM models to identify target terms.

**Fig 4:**
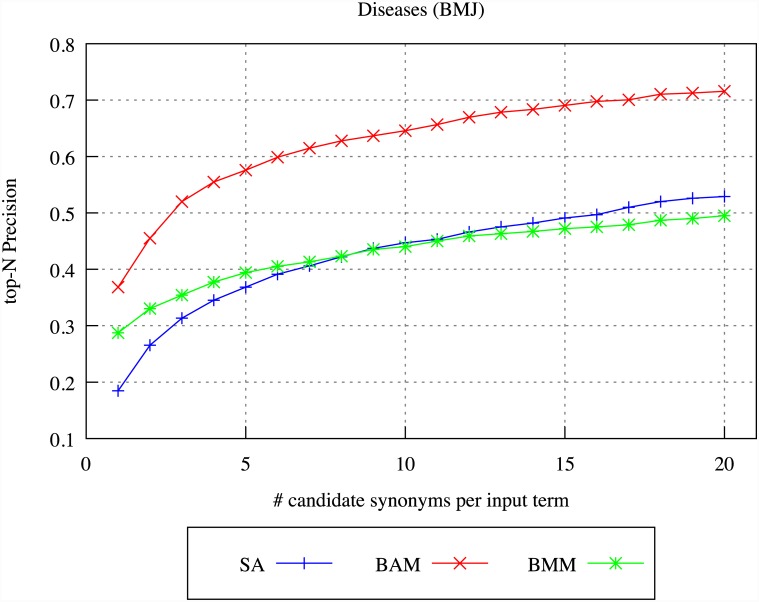
DSM precision for diseases in BMJ.

**Fig 5:**
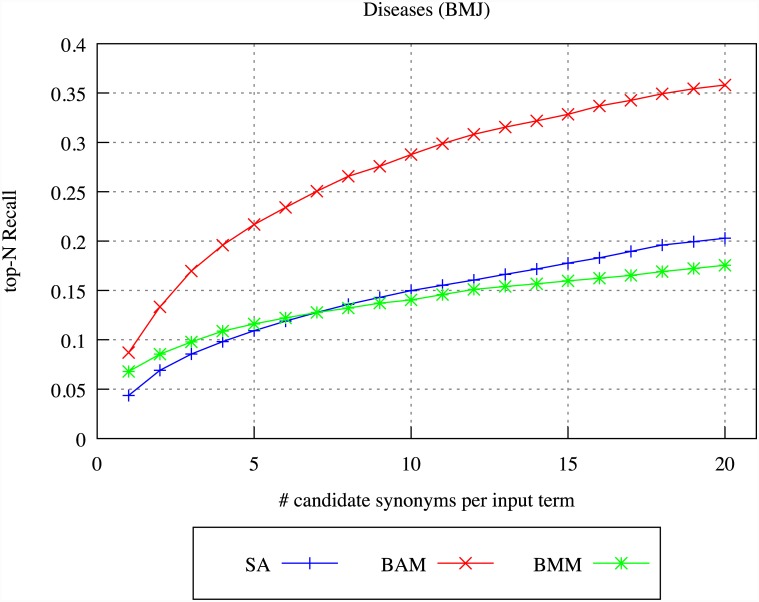
DSM recall for diseases in BMJ.

**Fig 6:**
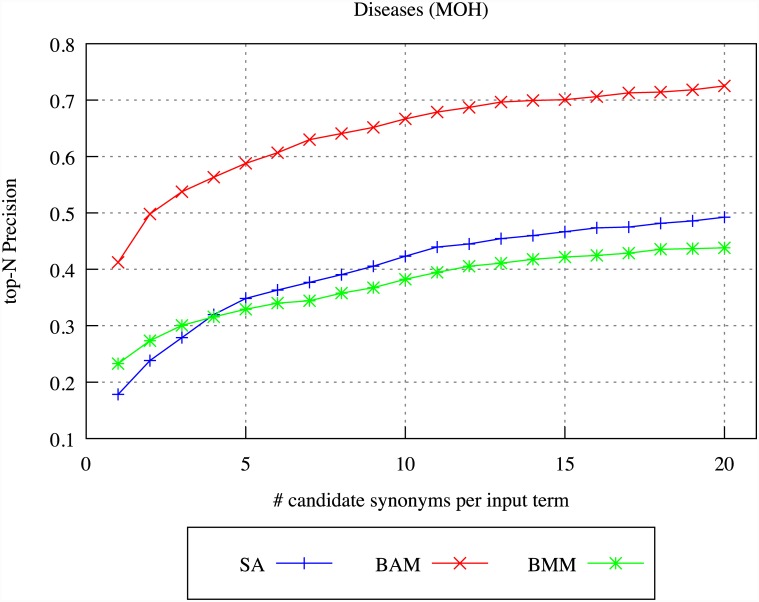
DSM precision for diseases in MOH.

**Fig 7:**
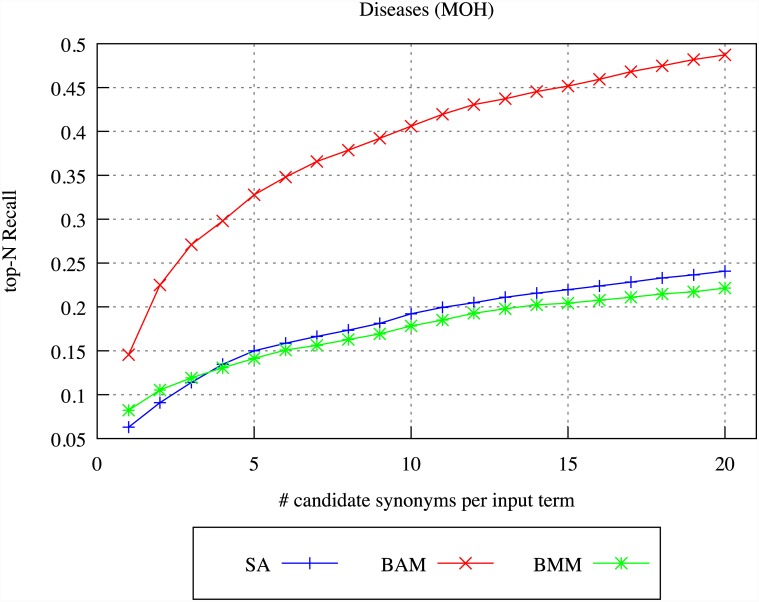
DSM recall for diseases in MOH.

Figs 4–7 illustrate our results for the different models in terms of precision and recall, considering differing numbers of top-ranked related terms, ranging from 1 up to 20. Precision is calculated by determining whether *any* of the top-ranked target terms identified by the indicated DSM is a UMLS synonym, whilst recall is calculated by determining *how many* of the UMLS synonyms of the source term appear within the top-ranked target terms identified by the DSM.

The BAM model performs considerably better than the other two approaches, in terms of both precision and recall, and for both archives. Given that a large number of multi-word terms occur within our evaluation data, this result provides clear evidence that exploiting the principle of compositionality can be extremely advantageous when dealing with multi-word terms, compared to when such terms are treated as single units (i.e., in the SA model). Although, as has been explained above, BMM is expected to produce better results than BAM when applied to sufficiently large datasets, in our case, the BMM results are significantly poorer than BAM. This suggests that, even though our archives are quite large, far vaster volumes of data are required for BMM to perform well.

The increase in precision obtained by considering a larger number of top-ranked related terms tends to become less marked after about 10 terms for both archives, i.e., if the model finds a UMLS synonym of a source term, it will usually correspond to one of the ten most similar target terms. For recall, there appears to be slightly more advantage in considering a greater number of related terms. In the MOH, when only a single top-ranked related term is considered, the recall is around 15%, partly as a consequence of many UMLS terms having more than one synonym; to achieve maximum recall, all synonymous terms listed in UMLS must be found. The greatest rate of recall increase, as more related target terms are considered, is observed for BAM, which achieves a recall of approximately 49% (for MOH) when 20 terms are considered. The recall for the top 20 terms in the BMJ is, however, rather lower than for the MOH. This could be related to the much smaller size of the MOH, meaning that fewer potentially related terms are generated, and thus there is a greater chance that all UMLS synonyms will be discovered.

#### Expert evaluation

The extent to which DSMs can identify related terms not present in UMLS (e.g., historically relevant synonyms and terms that are semantically related to the source term in other ways than synonymy) was determined via an evaluation by a medical historian. The evaluation involved 348 source-target term pairs identified by the BAM method, whose context vectors had a cosine similarity of 0.8 or greater, indicating that their textual contexts are very similar to each other. The evaluator was asked to assign one of the following categories to each source-target term pair:

- **Synonym**–diseases are (near) synonyms.
- **isA**–First disease is a subtype of the second.
- **isParent**–First disease is a supertype of the second.
- **Affects**–First disease has an effect on the second term.
- **IsAffectedBy**–First disease is affected by second term.
- **SpatiallyRelated**–Both diseases affect the same anatomical region.
- **OtherRelation**–Terms are related, but in a different way to any of the above classes.
- **Unrelated**–Both terms are diseases, but no semantic relation holds between them.
- **Non-disease pair**–The target term is not a disease.

The high correlation between pairs judged as synonyms according to UMLS, and those judged as synonyms by the expert, i.e., 0.8 Kappa [83] (a common means of calculating inter-annotator agreement when the task is to assign mutually exclusive, discrete categories to a set of annotation targets), provides strong evidence of the reliability of the expert decisions. An overview of the complete set of categorisations chosen by the evaluator is shown in Table 9.

**Table 9:** Expert semantic categorisation of DSM output.

Relation Type	Count (% proportion of total pairs)
**Synonym**	83 (24%)
**isA**	21 (6%)
**isParent**	35 (10%)
**Affects**	10 (3%)
**isAffectedBy**	11(3%)
**Spatially Related**	19 (5%)
**OtherRelation**	35 (10%)
**TOTAL SEMANTICALLY RELATED TERMS**	216 (62%)
**Unrelated**	106 (30%)
**Non-disease pair**	26 (7%)

Compared to the use of the UMLS Metathesaurus, which allowed 63 of the pairs (i.e., 18%) to be identified as synonyms, the expert identified 20 additional synonymous source-target term pairs (6%), yielding 83 (24%) pairs, and thus providing evidence that the BAM method can reliably identify synonyms not present in UMLS. Furthermore, a total of 62% of term pairs were determined to be semantically related in some way, which helps to demonstrate that DSMs can be useful in identifying various other types of semantic relations between terms. Whilst synonymy represents the most common relation, hierarchical relations are also quite frequently uncovered, and spatial relations are not uncommon. It is also significant that only in 7% of the pairs does the target term *not* represent a disease, i.e., the BAM model is very accurate in detecting target terms belonging to the correct semantic class.

Based upon the favourable evaluation results reported above, we applied the BAM model to the entire archives to create our temporal terminological inventory. In this case, however, the source terms used (around 175,000) corresponded to NEs of all seven categories identified by our TM pipeline that occur five or more times in the archives.

#### Semantic search system

As a demonstration of the utility of both the NEs and events extracted by our TM pipeline, and the information present within our automatically generated terminological inventory, they have been used as the basis for creating a semantic History of Medicine (HOM) search system (http://nactem.ac.uk/hom/) [84] to allow the exploration of information in both of the archives. The system aims to demonstrate how semantic information can be used to create a powerful, intuitive search interface that provides a number of extensions to standard keyword-based search systems, as follows:

- Exploration and comparison of the usage of related medical terms over time, through graphical visualisation.
- Expansion of search results, through automatic suggestion of terms related to original query terms, using the term inventory.
- Rapid refinement of results based on both document metadata (author, date, etc.) and the presence of specific types of semantic information (both entities and events with specified participants) within documents.
- Exploration of the semantic content of individual documents through highlighting of entities/events contained within them.

**Fig 8:**
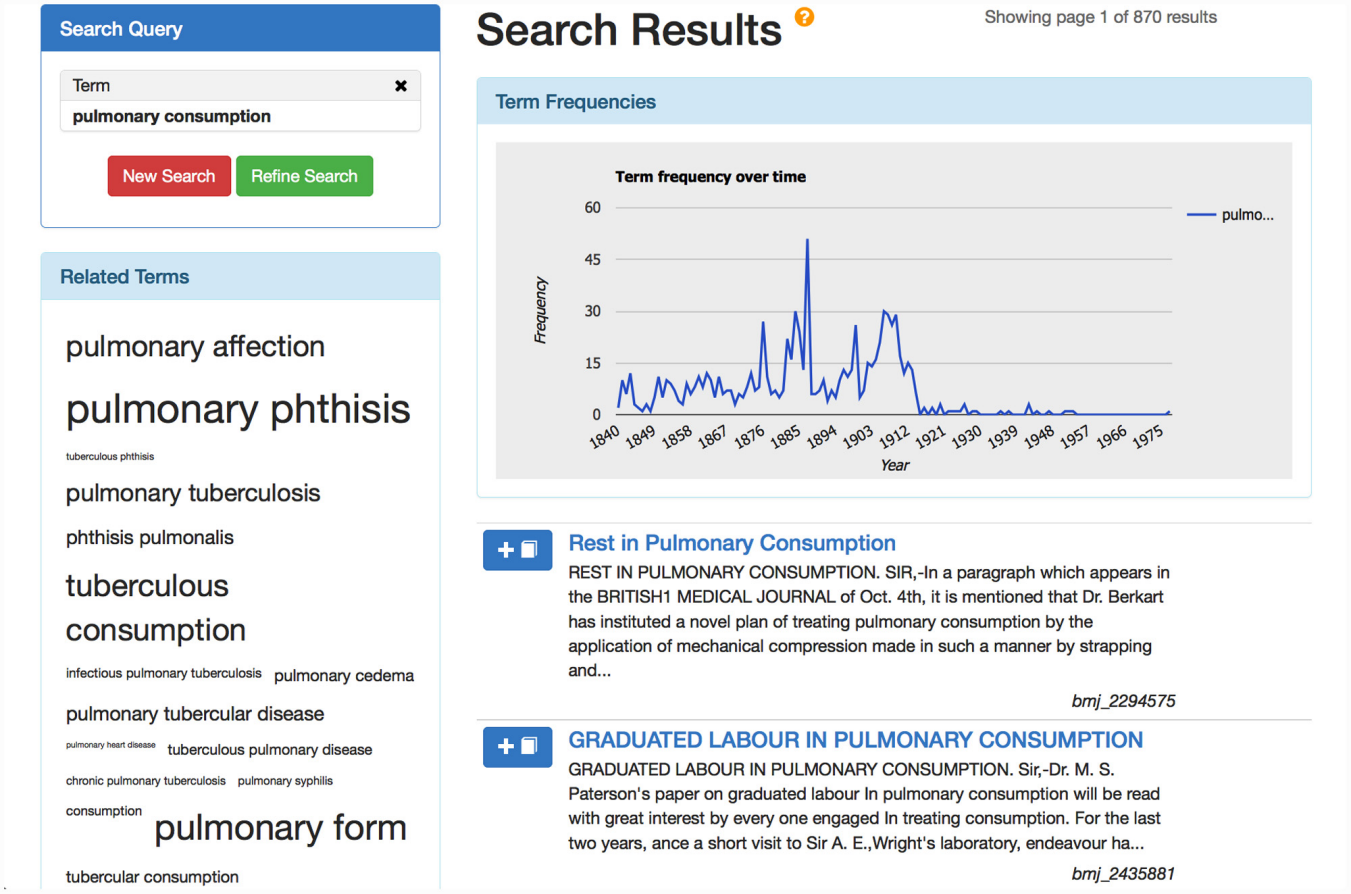
Term-based search in HOM.

Fig 8 shows a screenshot from the interface, in which the user has performed a search for the term *pulmonary consumption*. A graph illustrating the usage of this term over time shows that it becomes largely obsolete after about 1917. Related terms, whose size is determined according to their degree of contextual similarity to the user-entered term, may be clicked to rapidly expand queries. Graphs for any additionally selected terms will be superimposed, allowing their time specific usage to be compared.

**Fig 9:**
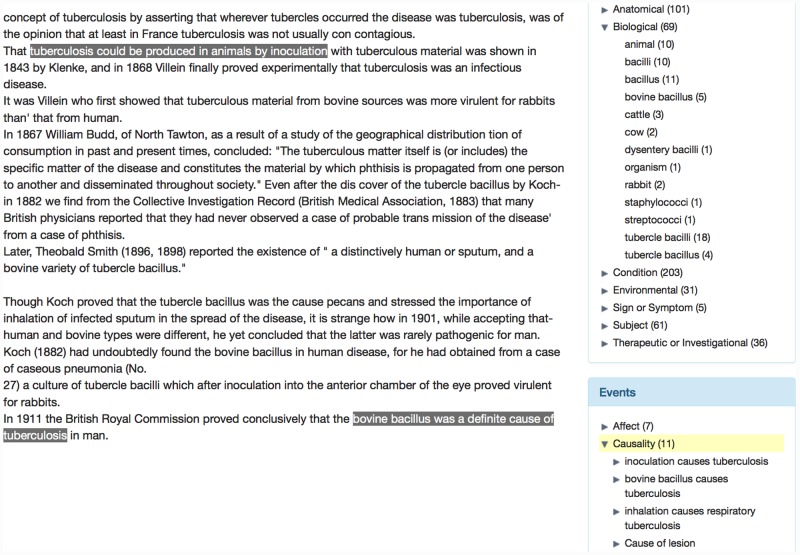
Individual document display in HOM.

Fig 9 shows the semantically enhanced display for a specific document. The right hand side of the screen provides information about the semantic information that has been recognised in the document. At the top, the exact numbers of the identified entities and event types are shown; each type may be expanded (by clicking on the arrow next to it) to reveal the specific information recognised within that category. In Fig 9, the *Biological* entity type and the *Causality* event type have been expanded; specific semantic information (entities or events) can be highlighted in the text by clicking on appropriate rows in the tree. This mechanism makes it easy to locate and examine the context of different types and amounts of information, depending on the task at hand.

In Fig 9, the *Causality* event type has been selected, such that all instances of this event type are highlighted in the text. Each textual highlight for an event corresponds to a text span that includes the trigger and all participants. It can be noticed from the highlighted text spans that different ways of expressing the causal event (here, the verb *produced* and the noun *cause*) are automatically recognised and mapped to the general causality semantic representation shown in the list of events within the *Events* tree on the right hand side of the screen. The ability to easily locate the relevant text spans can help to reveal greater contextual detail about their correct interpretation. For example, the highlighted event span at the bottom left of the figure specifies that bovine bacillus is *definitely* a cause of tuberculosis in man.

## Conclusions

This article has reported on the first attempt to create novel resources, i.e., a semantically annotated corpus, HIMERA, and a time-sensitive terminological inventory, to support the application of TM techniques to 19th and 20th century published medical text. The DSM used to construct the terminological inventory has been shown to be effective in detecting various semantic relationships between terms from different historical periods. Meanwhile, HIMERA has been used to train historically-focussed NE and event models, which have been integrated into a new TM pipeline for historical text. Our NE model has been shown to achieve robust, state-of-the-art performance across different text types and periods. We have also shown that, for the more complex task of event extraction, our model can identify useful relationships within the archives, and our on-going work to increase the size and scope of HIMERA will help to improve performance.

Our successful application of the TM pipeline to two large scale archives of digitised historical medical documents, the BMJ and the MOH, has shown the ability of the pipeline to robustly handle huge volumes of text. The power of the automatically extracted semantic information, especially when combined with the information contained within our temporal terminological inventory, has been demonstrated through their employment within a semantic search system that makes it easy for historians of medicine to explore and search the contents of the two archives efficiently, such that relevant documents from different periods may be retrieved straightforwardly, and answers to their research questions can be located rapidly.

Future extensions to HIMERA will include annotating both additional semantic types and information about the intended *interpretation* of events, so as to distinguish, e.g., definite from speculated events, the degree of speculation expressed, whether the information can be attributed to an information source other than the author, etc. We also intend to refine the terminological inventory, through both the application of more sophisticated DSMs, which use information such as NEs, syntactic dependencies and temporal information, and the investigation of automatic classification of the related terms identified, according to different types of semantic relations that they represent. Improvements to the search system will include automated clustering of documents according to semantic similarities, to increase the ease with which users can locate and explore the documents of the greatest relevance to them.
